# Exposure to environmental tobacco smoke among South Korean adults: a cross-sectional study of the 2005 Korea National Health and Nutrition Examination Survey

**DOI:** 10.1186/1476-069X-10-29

**Published:** 2011-04-03

**Authors:** Bo-Eun Lee, Eun-Hee Ha

**Affiliations:** 1 Department of Preventive Medicine and Medical Research Institute, School of Medicine, Ewha Womans University, 911-1 Mok-6-dong, Yangcheon-gu, Seoul, Korea

## Abstract

**Background:**

Studies have identified that environmental tobacco smoke exposure is associated with sociodemographic factors such as age, sex, and socioeconomic status, but few studies have been conducted in South Korea. In this study, the authors investigated the extent of environmental tobacco smoke exposure and factors related in a nationally representative sample of Korean adults.

**Methods:**

The data of 7,801 adults aged 19 years and over collected during the 2005 Korea National Health and Nutrition Examination Survey were analyzed. Information on smoking habits and exposure to environmental tobacco smoke was obtained by self-reports using a standardized questionnaire. Risks of environmental tobacco smoke exposure conferred by sociodemographic variables and behavioral risk factors were evaluated using logistic regression methods.

**Results:**

Overall, 36.1% of nonsmokers (defined as those not currently smoking) and 50.1% of current smokers were found to be exposed to environmental tobacco smoke either at work or at home. Among the nonsmokers, women were more likely to be exposed to environmental tobacco smoke at home (OR = 5.22, 95%CI, 4.08-6.67). Furthermore, an inverse relationship was found between education level and the risk of environmental tobacco smoke exposure at home (OR = 1.73, 95%CI, 1.38-2.17 for those with a high school education; OR = 2.30, 95%CI, 1.68-3.16 for those with a middle school education; and OR = 2.58, 95%CI, 1.85-3.59 for those with less than an elementary school education vs. those with a college education or more). In addition, those with office, sales service, or manual labor jobs were found to be at significantly higher risk of environmental tobacco smoke exposure at work than those with professional, administrative, or managerial jobs. Also, the risk of environmental tobacco smoke exposure in the workplace was significantly higher for alcohol drinkers than non-drinkers (OR = 1.23, 95%CI, 1.07-1.47). After adjusting for age, sex and education, it was found that those exposed to environmental tobacco smoke at home were more likely to have been admitted to hospital during the previous year (OR 1.29, 95%CI, 1.002-1.66).

**Conclusions:**

In this study of Korean adults, exposure to environmental tobacco smoke at home or work was found to be affected by sex, age, marital status, educational level, and type of occupation. Accordingly, these factors should be given appropriate consideration by those developing policies or interventions designed to control exposure to environmental tobacco smoke.

## Background

During the past decades, the male smoking rate in South Korea has reduced, but it is still high (45.0% in 2007) [1] and remains one of the highest among OECD (Organization for Economic Cooperation and Development) countries [2]. Furthermore, tobacco use among women and adolescents is increasing and the health effects of environmental tobacco smoke (ETS) have become a public health issue.

ETS, a Group 1 carcinogen, is composed of more than 4,000 chemicals [3]. Furthermore, the composition of sidestream smoke differs somewhat from mainstream smoke. Since the concentrations of the most harmful chemicals are higher in sidestream smoke, sidestream smoke could be more dangerous to health than mainstream smoke [4,5]. Many epidemiological and experimental studies have reported that exposure to ETS is associated with respiratory disease, cardiovascular disease, and cancer [6].

ETS exposure contributes to health inequalities and is a widespread health hazard [7]. Moreover, associations between ETS and health outcomes and the probably confounding effects of other risk factors, such as socioeconomic status, diet, and exposure to occupational carcinogens, have caused scientific and public concern [8]. Previous studies have suggested that ETS exposure is related to various factors, such as age, sex, socioeconomic status, and health risk behavior. However, few studies have assessed the strengths of associations between these variables and ETS exposure at home or in the workplace in Korea.

To formulate an effective policy and to devise preventive measures regarding the control of ETS, it is crucial that we understand the factors related to the prevalence of ETS exposure and identify target populations [9]. However, little data are available concerning the characteristics of ETS exposure in the general adult Korean population. Accordingly, the aim of this study was to determine the extent of ETS exposure and to identify related factors in a representative sample of Korean adults.

## Methods

### Study sample

We used data from the third Korea National Health and Nutrition Examination Survey (KNHANES III) [10], which was conducted in 2005 by the Korean Ministry of Health and Welfare. The survey consisted of the following components: a Health Survey (Health Interview Survey and Health Behavior Survey), a Health Examination Survey, and a Nutrition Survey. To achieve a nationally representative sample, a stratified multistage probability sampling method was used based on information on local government regions and dwelling type. There were approximately 246,000 primary sampling units, each of which contained approximately 60 households. For the Health Interview Survey, six hundred sampling frames (12,000 households) from the primary sampling units were randomly selected throughout South Korea, and of the six hundred sampling frames, two hundred sampling frames (4,000 households) were randomly selected for the Health Behavior Survey, the Health Examination Survey, and the Nutrition Survey.

The 2005 Health Behavior Survey involved the interviewing of 9,516 males and females aged 12 and over and had a response rate of 92.8%. The analysis reported in this article was restricted to 7,801 adults aged 19 and over who participated in the Health Behavior Survey. For the ETS at work analysis, we included only participants with a job at the time of survey.

### Data collection

#### Self-reported ETS exposure

The questionnaire included questions on sociodemographic variables, such as age, education, and marital status, lifestyle factors, such as alcohol drinking and exercise, and exposure to ETS in the home and at work.

We obtained information on smoking habits and ETS exposure from responses to self-assessment questions in the questionnaire. Current smokers were defined as those who had smoked at least 100 cigarettes in their lifetimes and who smoked on a daily basis at the time of the survey. Participants other than current smokers were defined as non-smokers, that is, they included never smokers and ex-smokers. Participants were asked to rate ETS exposure separately at home and work. They were classified as being exposed to ETS at home if any household member smoked at home, and as being exposed to ETS at work if they could smell tobacco smoke due to other people's smoking at work. In addition, all individuals that reported exposure to ETS at home or work were treated as have been exposed to ETS (any ETS group).

#### Sociodemographic indicators and behavioral risk factors

We used the following socio-demographic variables: sex, age (19-29, 30-39, 40-49, 50-59, 60-69, and ≥70), location of residence (urban/rural), and marital status (never married, married, and others). To characterize socioeconomic status, we used three variables, namely, education, household income, and occupation. We classified education level as: less than elementary (≤6 years in full-time education), middle (7-9 years), high (10-12 years), and college and above (>12 years). We established four income groups according to income quartiles. The lowest quartile was defined as the population with the lowest household equivalent income in 2005. Occupations were allocated to the following categories: legislator, senior officials and manager/professionals/technicians and associate professionals; clerks; service and sales workers; skilled agricultural, forestry and fishery workers; craft and related trade workers/plant, machine operators, and assemblers/manual laborers; military personnel; students; housewives; and unemployed.

Regarding behavioral risk factors, we included alcohol consumption (less than once a month, at least once a month), regular exercise during leisure time (no, yes), stress (low or moderate, high), rest (sufficient, insufficient), and health examination during the past 2 years (no, yes).

In the present study, health care utilization was used as a health outcome. Respondents are asked about hospitalization during the previous year, and about physician or pharmacy visits two weeks prior to the survey.

#### Statistical analysis

The age-specific prevalence of ETS exposure was calculated for males and females. The chi-square test was used to compare the prevalence of ETS exposure with respect to the various variables. To estimate the risks of ETS exposure by sociodemographic variables, we calculated unadjusted odds ratios (OR) and adjusted odds ratios (aOR) for exposure to ETS at home and work using logistic regression methods adjusted for all sociodemographic variables (sex, age, education, income, location of residence, marital status, and occupation). To examine the risk of ETS exposure with respect to behavioral risk factors, we performed multivariate analyses and adjusted for sex, age, and education. Statistical significance was accepted for p values of <0.05 throughout, and all analyses were performed using SAS ver. 8.2.

## Results

The general characteristics of the study subjects are provided in table 1. Fifty five percent of participants were female and 33.8% were graduates of at least high school level. Of the male subjects, 19.8% were never-smokers, 28.5% were former smokers and 51.7% were current smokers. Most women were never-smokers (91.5%).

**Table 1:** General characteristics of the study subjects from the 2005 Korea National Health and Nutrition Examination Study

	Male		Female		Total	
	**n**	**%**	**n**	**%**	**n**	**%**

**Sex**						

Male					3509	45.0

Female					4292	55.0

**Age groups (years)**						

19-29	583	16.6	753	17.5	1336	17.1

30-39	811	23.1	930	21.7	1741	22.3

40-49	872	24.9	1006	23.4	1878	24.1

50-59	579	16.5	650	15.1	1229	15.8

60-69	445	12.7	525	12.2	970	12.4

70+	219	6.2	428	10.0	647	8.3

**Education**						

College & above	526	15.3	1214	28.8	1740	22.7

High	376	10.9	476	11.3	852	11.1

Middle	1449	42.1	1594	37.8	3043	39.7

Elementary & less than	1093	31.7	935	22.2	2028	26.5

**Income**						

1(highest)	901	26.0	1097	25.8	1998	25.9

2	890	25.7	1097	25.8	1987	25.8

3	830	24.0	1028	24.2	1858	24.1

4(lowest)	843	24.3	1028	24.2	1871	24.3

**Location of residence**						

Rural	715	20.4	849	19.8	1564	20.1

Urban	2794	79.6	3443	80.2	6237	80.0

**Marital status**						

Never married	746	21.3	672	15.7	1418	18.2

Married	2533	72.3	2781	64.8	5314	68.2

Others	227	6.5	836	19.5	1063	13.7

**Smoking Status**						

Non-smoker	696	19.8	3929	91.5	4625	59.3

Ex-smoker	999	28.5	123	2.9	1122	14.4

Current smoker	1814	51.7	240	5.6	2054	26.3

Table 2 shows the prevalence of ETS exposure, according to current smoking status. Eighteen point three percent of nonsmokers were exposed to ETS at home, and 45.8% of those with a job were exposed to ETS at their workplace. The prevalence of persons who reported exposure to ETS either at home or at work was 36.1%. Men in their 20s had the highest rate of ETS exposure at home, and women in their 20s to 50s were significantly exposed to ETS at home. In terms of exposure to ETS at work, men showed a difference with respect to age and women in their 40s to 50s showed a significantly higher rate than other women. Both men and women showed significant differences to any ETS exposure by age.

**Table 2:** Prevalence of environmental tobacco smoke exposure according to status of current smoking in 2005 Korea National Health and Nutrition Examination Study

	Non-smoker						Current Smoker					
	**ETS exposure in home**		**ETS exposure in workplace**		**Any ETS exposure**		**ETS exposure in home**		**ETS exposure in workplace**		**Any ETS exposure**	
	**N**	**%**	**N**	**%**	**N**	**%**	**N**	**%**	**N**	**%**	**N**	**%**

**Total**	5747	18.3	2638	45.8	5747	36.1	2054	16.0	1333	62.6	2054	50.1

**Male**	1695	7.0	1035	54.2	1695	38.6	1814	14.1	1216	64.3	1814	51.3

**Age**												

19-29	258	20.3	120	55.0	258	40.0	325	22.6	202	64.9	325	51.8

30-39	327	3.1	300	54.0	327	50.7	484	13.4	424	66.3	484	64.0

40-49	387	1.9	310	57.5	387	47.5	485	8.4	377	63.0	485	52.0

50-59	299	4.5	192	52.9	299	37.0	280	13.6	156	60.6	280	41.1

60-69	273	4.0	97	43.9	273	19.6	172	11.1	50	64.3	172	28.3

70+	151	5.6	16	46.4	151	8.7	68	10.1	7	68.5	68	16.0

P-value^*^	<.0001		0.04		<.0001		<.0001		0.40		<.0001	

**Female**	4052	23.6	1603	39.9	4052	34.9	240	37.4	117	52.3	240	53.2

**Age**												

19-29	712	25.4	360	33.9	712	37.4	41	39.5	25	56.5	41	63.0

30-39	889	25.9	413	36.5	889	37.4	41	41.5	23	53.0	41	52.7

40-49	953	26.2	510	45.9	953	42.7	53	53.8	34	61.8	53	79.8

50-59	608	23.7	222	46.7	608	36.5	42	24.4	26	38.6	42	38.6

60-69	506	15.4	86	34.4	506	19.6	19	19.5	7	41.0	19	27.1

70+	384	15.2	12	30.2	384	16.0	44	26.8	2	0.0	44	26.8

P-value^*^	<.0001		0.003		<.0001		0.04		0.18		<.0001	

* Significance determined using χ^2 ^test of trends

It was also observed that the smokers were more exposed to ETS at work than non-smokers. In terms of any ETS exposure, men and women showed significant differences according to age. A higher rate of any ETS exposure was found among young smokers in their 20s to 40s.

Table 3 shows the risks of exposure to ETS according to sociodemographic factors. Among nonsmokers, women were 5.22 times more likely to be exposed to ETS at home than men. Furthermore, subjects in their 20s, 30s, and 40s were more likely to be exposed ETS at home than those in 70s. In addition, high school graduates were found to be 1.73 times more likely to be exposed to ETS than college graduates or those with a higher education. Furthermore, for middle school graduates, and elementary school graduates or those with lower levels of educational achievement, the odds ratios were 2.30 (95%CI, 1.68-3.16) and 2.58 (95%CI, 1.85-3.59), respectively, indicating an inverse relationship between educational attainment and the risks of ETS exposure. It was also observed that those who were divorced or widowed were less likely to be exposed to ETS at home than never married respondents (OR = 0.36, 95%CI, 0.24-0.53). However, no significant difference in exposure to ETS at home was found with respect to income or location of residence. On the other hand, those with office or sales service jobs, simple laborers, housewives, and the unemployed were found to be at higher risk of being exposed to ETS at home than those with professional, administrative, or managerial jobs. Regarding ETS exposure in the workplace, women were less likely to be exposed to ETS than men (OR = 0.44, 95%CI, 0.37-0.53). Furthermore, lower educational attainment was found to be associated with a higher risk of ETS exposure in the workplace. In particular, those with office or sales service jobs, or manual labor jobs had a significantly higher risk of ETS exposure at the workplace than those with professional, administrative, or managerial jobs. As was found for non-smokers, for smokers, a higher exposure to ETS at home was found among women, those with a low level of education, and those that had never married. Furthermore, smoker exposure to ETS at work was found to differ by sex, age, and type of occupation.

**Table 3:** Risks of environmental tobacco smoke exposure according to sociodemographic factors

	Non Smoker				Current Smoker			
	**ETS exposure in home**		**ETS exposure in workplace**		**ETS exposure in home**		**ETS exposure in workplace**	
	**OR (95% CI) **^**b**^	**AOR**^**a **^**(95% CI)**	**OR (95% CI)**	**AOR**^**a **^**(95% CI)**	**OR (95% CI)**	**AOR**^**a **^**(95% CI)**	**OR (95% CI)**	**AOR**^**a **^**(95% CI)**

**Sex**								

Male	1.00	1.00	1.00	1.00	1.00	1.00	1.00	1.00

Female	5.29 (4.24-6.61)	5.22 (4.08-6.67)	0.59 (0.50-0.69)	0.44 (0.37- 0.53)	3.69 (2.74-4.96)	3.62 (2.38-5.50)	0.60 (0.41-0.88)	0.62 (0.42-0.91)

**Age (years)**								

19-29	2.13 (1.58-2.87)	2.60 (1.61-4.19)	1.01 (0.46-2.20)	1.94 (0.76-4.90)	1.71 (0.97-3.03)	1.69 (0.71-3.99)	2.04 (0.53-7.84)	10.20 (2.86-36.37)

30-39	1.78 (1.33-2.40)	2.07 (1.35-3.16)	1.23 (0.56-2.66)	1.94 (0.80-4.67)	1.005 (0.56-1.77)	1.54 (0.68-3.47)	2.37 (0.62-8.98)	11.47 (3.31-39.76)

40-49	1.70 (1.27-2.28)	1.65 (1.10-2.46)	1.48 (0.68-3.21)	1.86 (0.78-4.41)	0.82 (0.46-1.46)	1.30 (0.60-2.82)	2.10 (0.55-7.96)	8.33 (2.43-28.50)

50-59	1.57 (1.15-2.14)	1.32 (0.90-1.92)	1.53 (0.70-3.34)	1.51 (0.63-3.57)	1.07 (0.59-1.95)	1.42 (0.69-2.94)	1.59 (0.41-6.12)	5.52 (1.62-18.81)

60-69	0.96 (0.69-1.35)	0.85 (0.58-1.24)	1.02 (0.45-2.31)	0.94 (0.38-2.28)	0.84 (0.43-1.63)	1.20 (0.56-2.54)	2.14 (0.51-8.87)	4.18 (1.19-14.66)

70+	1.00	1.00	1.00	1.00	1.00	1.00	1.00	1.00

*P *for trend	<.0001	<.0001	0.07	0.001	0.006	<.0001	0.16	0.01

**Education**								

College & above	1.00	1.00	1.00	1.00	1.00	1.00	1.00	1.00

High	1.98 (1.64-2.39)	1.73 (1.38-2.17)	1.81 (1.52-2.17)	1.59 (1.27-1.98)	1.68 (1.22-2.32)	1.39 (0.94-2.06)	0.88 (0.71-1.09)	0.69 (0.53-0.91)

Middle	1.88 (1.47-2.40)	2.30 (1.68-3.16)	2.46 (1.89-3.21)	2.54 (1.80-3.57)	2.11 (1.37-3.25)	2.12 (1.22-3.67)	0.64 (0.46-0.89)	0.70 (0.45-1.07)

Elementary & less than	1.52 (1.23-1.87)	2.58 (1.85-3.59)	1.44 (1.11-1.87)	1.85 (1.28-2.68)	1.69 (1.14-2.51)	1.79 (0.99-3.22)	0.27 (0.19-0.37)	0.65 (0.40-1.03)

*P *for trend	0.007	<.0001	<.0001	0.0003	0.005	0.01	0.06	0.16

**Income**								

1(highest)	1.00	1.00	1.00	1.00	1.00	1.00	1.00	1.00

2	1.27 (1.04-1.54)	1.04 (0.84-1.29)	1.07 (0.87-1.32)	0.89 (0.72-1.11)	1.16 (0.80-1.66)	1.009 (0.68-1.48)	0.84 (0.64-1.10)	0.71 (0.52-0.96)

3	1.38 (1.13-1.67)	1.09 (0.88-1.34)	1.25 (1.01-1.54)	0.97 (0.77-1.22)	1.07 (0.75-1.52)	0.86 (0.58-1.27)	0.82 (0.63-1.06)	0.67 (0.49-0.91)

4(lowest)	1.30 (1.07-1.58)	0.99 (0.79-1.23)	1.35 (1.07-1.70)	0.98 (0.76-1.27)	1.02 (0.72-1.44)	0.71 (0.48-1.06)	0.55 (0.42-0.71)	0.51 (0.37-0.69)

*P *for trend	0.005	0.83	0.003	0.81	0.91	0.06	0.09	0.94

**Location of residence**								

Rural	1.00	1.00	1.00	1.00	1.00	1.00	1.00	1.00

Urban	0.92 (0.78-1.09)	0.87 (0.72-1.07)	1.16 (0.93-1.45)	1.19 (0.93-1.51)	1.03 (0.77-1.38)	1.04 (0.74-1.47)	1.15 (0.84-1.56)	0.74 (0.56-0.99)

**Marital status**								

Never married	1.00	1.00	1.00	1.00	1.00	1.00	1.00	1.00

Married	0.91 (0.76-1.09)	0.84 (0.62-1.16)	1.26 (1.04-1.53)	0.95 (0.70-1.30)	0.49 (0.37-0.63)	0.47 (0.32-0.71)	1.26 (1.02-1.57)	1.62 (1.17-2.23)

Others	0.47 (0.36-0.62)	0.36 (0.24-0.53)	1.17 (0.87-1.59)	0.93 (0.61-1.42)	0.44 (0.29-0.69)	0.22 (0.12-0.42)	0.44 (0.31-0.63)	0.96 (0.59-1.57)

**Occupation**								

Legislator, senior officials and manager,professionals,technicians and associate professionals	1.00	1.00	1.00	1.00	1.00	1.00	1.00	1.00

Clerks	2.10 (1.43-3.11)	1.50 (1.001-2.27)	1.88 (1.46-2.42)	1.83 (1.40-2.40)	1.07 (0.56-2.03)	1.01 (0.52-1.98)	4.50 (3.21-6.31)	2.63 (1.82-3.78)

Service and sales workers	3.01 (2.12-4.28)	1.98 (1.33-2.94)	2.74 (2.18-3.46)	2.52 (1.91-3.33)	1.90 (1.12-3.24)	1.28 (0.68-2.38)	8.77 (6.53-11.78)	8.02 (5.75-11.16)

Skilled agricultural, forestry and fishery	1.95 (1.28-2.97)	1.52 (0.92-2.50)	0.77 (0.36-1.68)	0.71 (0.31-1.62)	1.18 (0.57-2.46)	1.20 (0.50-2.87)	0.20 (0.08-0.51)	0.35 (0.13-0.93)

Craft and related trades workers/Plant, machine operators and Assemblers/Manual laborer	2.66 (1.87-3.78)	2.28 (1.51-3.43)	2.57 (2.03-3.24)	1.86 (1.39-2.49)	1.80 (1.09-2.94)	1.55 (0.85-2.80)	5.33 (4.17-6.81)	5.45 (4.06-7.31)

Students	2.78 (1.80-4.30)	1.24 (0.74-2.08)	-	-	2.34 (1.10-4.99)	0.93 (0.37-2.29)	-	-

Housewives	3.79 (2.72-5.27)	1.89 (1.29-2.76)	-	-	18.76 (8.86-39.71)	6.34 (2.54-15.85)	-	-

Unemployed	1.50 (1.03-2.18)	1.78 (1.15-2.77)	-	-	1.81 (1.06-3.08)	1.58 (0.82-3.02)	-	-

^a ^Adjusted for sex, age, education, income location of residence, marital status, and occupation

^b ^OR, odds ratio; CI, confidence interval

The risks of exposure to ETS according to health behavior factors are shown in Table 4. Among nonsmokers, the risks of ETS exposure at home were higher for those who drank alcohol regularly, did not exercise regularly, were under heavy stress, and who undergo regular health examinations. However, the confidence interval was not significant. On the other hand, the risk of exposure to ETS in the workplace was significantly higher for alcohol drinkers (OR = 1.25, 95%CI, 1.07-1.47) than non-drinkers. In the case of current smokers, no meaningful relation was found between ETS exposure at home and at the workplace and health behavior. Those who had undergone a health examination during the two previous years were found to have a significantly lower ETS exposure risk than those who did not (OR = 0.77; 95%CI, 0.61-0.98).

**Table 4:** Risks of environmental tobacco smoke exposure according to health behavior factors

	Non-smoker				Current Smoker			
	**ETS exposure in home**		**ETS exposure in workplace**		**ETS exposure in home**		**ETS exposure in workplace**	
	**OR (95%CI)**	**AOR (95%CI)**	**OR (95%CI)**	**AOR (95%CI)**	**OR (95%CI)**	**AOR (95%CI)**	**OR (95%CI)**	**AOR (95%CI)**

**Experience of alcohol drinking**								

No	1.00	1.00	1.00	1.00	1.00	1.00	1.00	1.00

Yes	1.09 (0.95-1.24)	1.09 (0.95-1.26)	1.23 (1.06-1.44)	1.25 (1.07-1.47)	0.88 (0.67-1.15)	1.06 (0.79-1.44)	1.17 (0.89-1.54)	1.16 (0.87-1.54)

**Regular exercise**								

Yes	1.00	1.00	1.00	1.00	1.00	1.00	1.00	1.00

No	1.05 (0.91-1.21)	1.07 (0.92-1.25)	0.88 (0.75-1.04)	0.87 (0.74-1.03)	1.18 (0.89-1.54)	1.08 (0.81-1.45)	1.28 (1.00-1.64)	1.16 (0.89-1.50)

**Stress**								

Low/Moderate	1.00	1.00	1.00	1.00	1.00	1.00	1.00	1.00

High	1.02 (0.89-1.17)	1.02 (0.88-1.19)	1.02 (0.87-1.20)	1.04 (0.88-1.23)	0.89 (0.70-1.14)	0.87 (0.67-1.13)	1.29 (1.03-1.61)	1.24 (0.97-1.57)

**Rest**								

Sufficient	1.00	1.00	1.00	1.00	1.00	1.00	1.00	1.00

Insufficient	0.85 (0.74-0.99)	0.82 (0.70-0.95)	0.97 (0.83-1.14)	0.94 (0.79-1.11)	0.83 (0.64-1.07)	0.81 (0.61-1.08)	1.27 (1.01-1.60)	1.13 (0.89-1.45)

**Health examination**								

No	1.00	1.00	1.00	1.00	1.00	1.00	1.00	1.00

Yes	1.04 (0.91-1.19)	1.08 (0.93-1.24)	0.90 (0.77-1.05)	0.89 (0.76-1.05)	0.64 (0.50-0.81)	0.85 (0.65-1.10)	0.75 (0.59-0.94)	0.77 (0.61-0.98)

Adjusted for sex, age, and education

**Figure 1:**
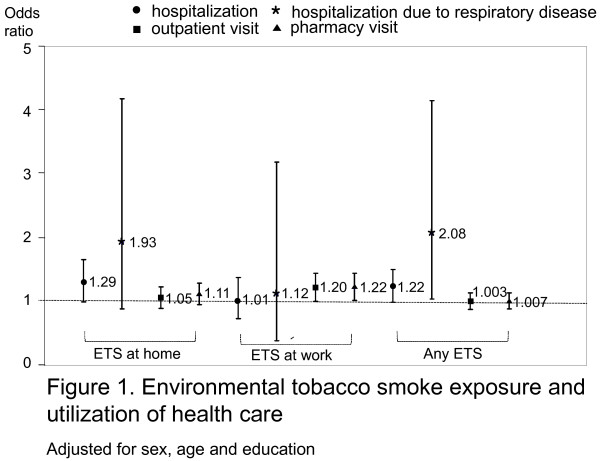
Environmental tobacco smoke exposure and utilization of health care

Figure 1 shows the relationship between ETS exposure and the utilization of health care services. Those who were exposed to ETS at home had a 1.29 times higher hospitalization rate over the previous year (95%CI, 1.002-1.661), and those exposed to ETS at home or work (i.e., any ETS) reported more hospitalization due to respiratory disease than those not exposed (OR = 2.08; 95%CI, 1.04-4.16). Also, those exposed to ETS at work visited outpatient departments and pharmacies 1.20 and 1.22 times more than those not exposed at work, respectively.

## Discussion

It was found in this study that 36.1% of Korean nonsmokers over 19 years of age were exposed to ETS at home or at work in 2005. Nonsmoker exposure to ETS at home was found to be associated with sex, age, education history, marital status, and type of occupation, and exposure to ETS at work with sex, education level, and type of occupation. In general, exposure to ETS did not show any significant relationship to health behavior, but those who drank more than once a month had a significantly higher ETS exposure risk at work. On the other hand, exposure to ETS was found to be related to the utilization of health care services, and in particular, exposure to ETS at home and ETS at work was found to be associated with hospitalization, and outpatient visits or pharmacy visits, respectively.

This research shows that 36.1% of the subjects were exposed to ETS at home or at work in Korea, which is less than the 49.2% reported in China [11], and the 69.5% for men and the 62.9% for women reported in Spain [12], and it is closer to that found in Cambodia (37.4%) [13]. However, the rate of ETS exposure in Finland was reported to be only 14.3% for male nonsmokers and 13% for female nonsmokers [14], and in America to be 20.2% for those who have never smoked [15]. These figures show that the prevalence of ETS exposure in Korea is still higher than that in developed countries. Therefore, political and social efforts are needed to decrease non-smoker ETS exposure.

This research also shows that in South Korea, ETS exposure in adult population at home is 18.3%, which is close to the 14% reported by a Norwegian study [16] and the 20% for men and 26% for women reported by an American study [17]. These prevalences are markedly lower than the 54.3% found in a Taiwanese study [18] and the 41.4% found in a Chinese study [11]. In the present study, ETS exposure at home was about four times higher for women (23.3%) than men (5.43%), which concur with previous studies [11-13,17,19]. This may be due to women spending more time at home and the fact that more spouses of female non-smokers smoke than spouses of male non-smokers. In particular, female non-smokers who live in overcrowed conditions are at greater risk of ETS exposure [6].

ETS exposure is not evenly distributed across the population, and it has been established that sociodemographic factors, such as education level and occupation, are related to exposure [20]. This study shows that the risks of ETS exposure at home increase as education level decreases, and at work that it is significantly higher for those with a low education level. Research in New Zealand also showed that nonsmoking adults with a low social and economic status were more exposed to ETS, and among the socioeconomic factors studied, education was found to be most related to ETS exposure [21]. According to a study by Iribarren et al. (2001), those exposed to ETS for more than 40 hours a week were likely to have no or only a partial college education [17]. Those who left school before the ninth grade were found to be twice as likely to be exposed to ETS at home than college graduates [9], and another study reported that those with 6 years or more of education were less likely to be exposed to ETS at home [13].

It has been reported that workers in lower socioeconomic groups are more likely to be exposed to ETS at work [7,22]. This research also found that those with office jobs, sales service jobs, and simple labor jobs have much higher rates of ETS exposure at work than those with professional, administrative, or managerial jobs. Moussa et al. reported that men who are engaged in skilled manual work and women who are engaged in unskilled manual work are more at risk of ETS exposure at work [7]. Because the prevalence of active smoking is higher among lower social classes, they are more likely to be exposed to ETS generated by their smoking coworkers [22].

Previous studies have also addressed the relationships between risk behaviors and exposure to ETS. Stamatakis et al. found that lack of cancer screening and inadequate fruit and vegetable consumption were associated with exposure to ETS at home and work [9]. Curtin et al. reported that female non-smokers with less healthy eating habits were more exposed to ETS at work [20], and in another study, women exposed to second-hand smoke at home were found to have less healthy eating habits and preventive screening practices [23]. In this study, we found a significant relationship between alcohol drinking and exposure to ETS at work, which concurs with a Spanish study that found a relationship between alcohol consumption and second-hand smoke exposure [12]. Therefore, it is important to consider various risk behaviors for such researches that are aimed at figuring out ETS and its effects on health.

Although ETS exposure is a diluted form of exposure, certain toxic and carcinogenic chemicals are present at higher proportions in sidestream smoke than in mainstream smoke [24]. Furthermore, it is plausible that ETS exposure could increase the risk of respiratory diseases in healthy adults. Ostro (1989) found that ETS was significantly associated with respiratory morbidity in adults after adjusting for air pollution and active smoking [25]. In addition, ETS exposure has been reported to increase the risks of chronic infections of the lower respiratory tract in adults and children [26]. A study conducted in Hong Kong found that male police officers exposed to passive smoking for more than a year had 1.36 times more consultations with doctors due to respiratory problems during the previous 14 days and 1.79 times more medication use due to respiratory problems than those not exposed [27]. In addition, we found that hospitalization due to respiratory disease was higher among never-smokers exposed to any ETS than among never-smokers not exposed to any ETS (OR = 2.08, 95%CI, 1.04-4.16).

In the present study, we also found that ETS exposure at home is related to hospitalization during the previous year and that ETS exposure at work is related to an outpatient visit and pharmacy visit during the previous two weeks. The results of our study are supported by the results of previous studies, which found that exposure to ETS has adverse effects on health, as indicated by self-reported health conditions [15,17], restricted activity [15], and acute and chronic medical conditions, such as asthma and cold/flu symptoms [17].

The strength of the present study is that we were able to characterize current ETS prevalence in relation to sociodemographic factors, based on a representative sample of the Korean population using KNHANES data. However, this study has some limitations. First, the cross sectional design of this study does not allow inference of causality. Also, cross-sectional data based on questionnaire responses could cause some degree of systematic error [12]. Second, retrospective self-reports of ETS exposure could be prone to errors, such as misreporting or recall bias. However, we did not test subjects with biological markers or obtain detailed information regarding the frequency and amount of ETS exposure, and thus, it is difficult to assess the validity of self-reported ETS data. Nevertheless, it has been reported that self-reported ETS data are sufficiently valid to provide population estimates [28-30]. Third, we did not collect information on ETS exposure during leisure time, which could be a substantial source of ETS exposure among the young [31].

## Conclusions

The smoking rate in Korea remains higher than in any other country [2]. This study shows that about one in three Korean nonsmoking adults is exposed to ETS. Whereas men were found to be most exposed to ETS at work, women were found to be most exposed at home. Furthermore, a low socioeconomic status, as indicated by a low education level or occupational status, was found to be associated with greater ETS exposure at home and work. This study shows that adults who have never smoked, but who are exposed to ETS, more frequently visit medical facilities than those not exposed.

These findings imply that urgent action is required to lower the prevalence of smoking and to limit exposure to ETS in South Korea. To reduce exposure to ETS at workplaces and other public areas, health education regarding the risks of ETS should be given priority. In addition, consideration of socioeconomic factors, such as education level, is required when considering policy or intervention options designed to control exposure to ETS.

## List of Abbreviations

ETS:Environmental Tobacco Smoke; KNHANES:Korea National Health and Nutrition Examination Survey; OECD:Organization for Economic Cooperation and Development; OR:Odds Ratio.

## Authors' contributions

BEL reviewed the literature, analyzed the data and wrote the manuscript. EHH directed the whole study and provided feedback and guidance on data analysis and manuscript drafts. All authors read and approved the final manuscript.

## Competing interests

The authors declare that they have no competing interests.
